# Clinical and radiological analysis of the effects of three different lumbar transpedicular dynamic stabilization system on disc degeneration and regeneration

**DOI:** 10.3389/fsurg.2023.1297790

**Published:** 2023-12-15

**Authors:** Mehmet Kursat Karadag, Mehmet Yigit Akgun, Ahmet Tulgar Basak, Ozkan Ates, Mehmet Ali Tepebasili, Caner Gunerbuyuk, Tunc Oktenoglu, Mehdi Sasani, Ali Fahir Ozer

**Affiliations:** ^1^ Department of Neurosurgery, Ataturk University, Erzurum, Türkiye; ^2^ Department of Neurosurgery, Koc University Hospital, Istanbul, Türkiye; ^3^ Spine Center, Koc University Hospital, Istanbul, Türkiye; ^4^ Department of Neurosurgery, American Hospital, Istanbul, Türkiye

**Keywords:** disc regeneration, disc degeneration, lumbar dynamic systems, lordosis angle, intervertebral disc

## Abstract

**Objective:**

This study aims to assess the clinical outcomes of three transpedicular dynamic systems in treating degenerative disc disease and evaluate their impact on both clinical and radiological aspects of the operated and adjacent segments.

**Materials and methods:**

A total of 111 patients who underwent posterior transpedicular short-segment dynamic system procedures for treatment of degenerative disc disease were included. The patients were categorized into three groups, namely, Group 1 (Dynesys system, *n* = 38), Group 2 (Safinaz screw + PEEK rod, *n* = 37), and Group 3 (Safinaz screw + titanium rod, *n* = 36). Disc regeneration in the operated segment and disc degeneration in the operated, upper, and lower adjacent segments were assessed using the Pfirrmann Classification.

**Results:**

Postoperatively, a statistically significant difference was observed in visual analog scale and Oswestry Disability Index scores (*p* < 0.001). However, no statistically significant difference was seen in disc degeneration/regeneration and degeneration scores of the upper and lower adjacent segments between the preoperative and postoperative groups (*p* = 0.763, *p* = 0.518, *p* = 0.201). Notably, a positive effect on disc regeneration at the operated level (32.4%) was observed. No significant differences were found between the groups in terms of operation rates, screw loosening, and screw breakage after adjacent segment disease (*p* > 0.05).

**Conclusion:**

In patients without advanced degeneration, all three dynamic systems demonstrated the ability to prevent degeneration in the adjacent and operated segments while promoting regeneration in the operated segment. Beyond inhibiting abnormal movement in painful segments, maintaining physiological motion and providing axial distraction in the operated segment emerged as key mechanisms supporting regeneration.

## Introduction

Lumbar degenerative disc diseases constitute a prevalent group of conditions that escalate in incidence with age, predominantly affecting the middle-aged demographic. These ailments not only compromise the quality of life but also contribute to significant economic burdens. Etiologically, aging, disc tissue malnutrition, trauma, pathological loads, and various epidemiological factors collectively play pivotal roles (1, 2).

The degeneration process initiates with a decline in proteoglycans, followed by a reduction in osmotic pressure, ultimately leading to dehydration. Consequently, the degenerated disc faces challenges in self-regeneration due to a compromised blood supply and heightened intradiscal pressure (3). The structural deterioration of the disc disrupts the equilibrium of load distribution along the spine, resulting in an increased load on the posterior elements. Pain ensues as a consequence of the imbalanced load distribution and abnormal movements induced by instability (4–6).

Fusion surgery has emerged as the predominant method for achieving stabilization, yielding satisfactory outcomes by thwarting segmental pathological movements. However, the long-term follow-up of patients undergoing fusion surgery reveals potential complications, such as pseudoarthrosis, flat back, implant fracture, implant loosening, and adjacent segment degeneration. These complications underscore the necessity for meticulous postoperative monitoring and tailored interventions to mitigate adverse effects and optimize patient outcomes (7–9).

The challenges associated with complications such as implant loosening and fracture have prompted researchers to explore alternative solutions, leading to the development of dynamic systems. These systems play a crucial role in achieving a more balanced distribution of load on the spine, with the aim of preventing implant-related issues (7, 10). Furthermore, by regulating impaired intradiscal pressure in the operated segment, these dynamic systems not only facilitate rehydration but also hold a promise in preventing degeneration progression and promoting regeneration (11–13).

This study delves into three distinct posterior pedicular dynamic systems utilized in treating single-level degenerative disc disease, shedding light on their nuances in the context of existing literature. The investigation seeks to unveil the impacts of these systems on the adjacent segment, the mechanics of the moving segment, and segmental regeneration. Following neural structure decompression, the application of these three dynamic stabilization systems is designed to preserve physiological segmental movement.

## Materials and methods

In this study, all procedures performed were in accordance with the ethical standards of the institutional and national research committee (Ataturk University Faculty of Medicine Clinical Research Ethics Committee, Date: 27 January 2022, Approval No: B.30.2.ATA.0.01.00/109) and with the 1964 Helsinki Declaration and its later amendments or comparable ethical standards. Informed consent was obtained from all participants included in the study. The Ethics Committee of Ataturk University Faculty of Medicine Clinical Research approved this study.

### Study design

Between 2008 and 2019, a total of 111 patients diagnosed with degenerative disc disease underwent treatment with one of three distinct posterior transpedicular dynamic stabilization systems. The study cohort comprised 70 females and 41 males. Short-segment stabilization was uniformly applied across all patients. The groups were stratified as follows:

Group 1: 38 patients (22 females, 16 males) treated with Dynesys DSS (Zimmer Inc., Warsaw, IN, USA).

Group 2: 37 patients (26 females, 11 males) treated with Safinaz DSS (Medikon Tipsan AS, Türkiye) and PEEK rod.

Group 3: 36 patients (22 females, 14 males) treated with Safinaz DSS and titanium rod.

The evaluation encompassed the operated segment and an upper segment in a cohort of 111 patients. Notably, the pathological segment was identified as L5–S1 in 36 patients, allowing for a sub-segment analysis involving 75 patients. It is imperative to recognize that distinct indications may apply to each specific segment.

The main indications for surgery persistence of pain despite conservative treatment, painful disc, annulus defect, Modic degeneration, and narrow canal. Appropriate nonsteroidal anti inflammatory drug (NSAID) treatment was initiated for the preoperative conservative treatment for approximately 3 to 4 weeks. In addition to medical treatment, the patients who did not have neurological deficits were also referred to a 15-session physical therapy and rehabilitation program. The patients with a history of spinal surgery, vertebral fractures, advanced osteoporotic vertebrae, spondylolisthesis, infection, and malignancy were excluded from the study.

### Dynamic systems

#### Dynesys system

**Figure 1:**
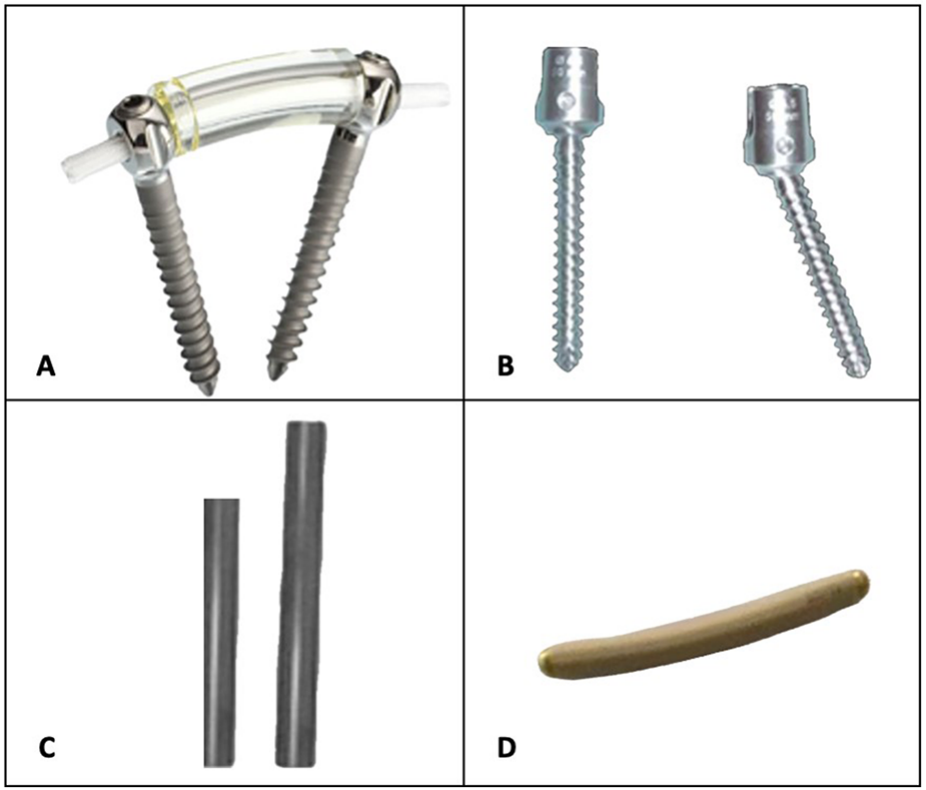
Dynamic systems: (**A**) Dynesys system. (**B**) Safinaz screw. (**C**) Ti rod. (**D**) PEEK rod.

The Dynesys system was manufactured by Zimmer Spine. This system was developed by Dubois in 1994. It is currently the most frequently used dynamic system in the world. In this system, an inelastic tension band is placed between the peduncle screws, and a polyurethane spacer is placed around the band. The screw is monoaxial transpedicular type. Not the screw, but the system is dynamic (Figure 1).

#### Safinaz screw

This dynamic screw was developed by Ali Fahir Ozer (Medikon, Ankara, Türkiye) based on the Cosmic system. It is a polyaxial transpedicular system that allows up to 20° of flexion–extension and 2° rotational movement. In our study, the Safinaz screw system, in which we used one of the groups with a PEEK rod and the other with a titanium rod, is available in the market and is widely used in our clinic (Figure 1).

### Radiological evaluation

**Figure 2:**
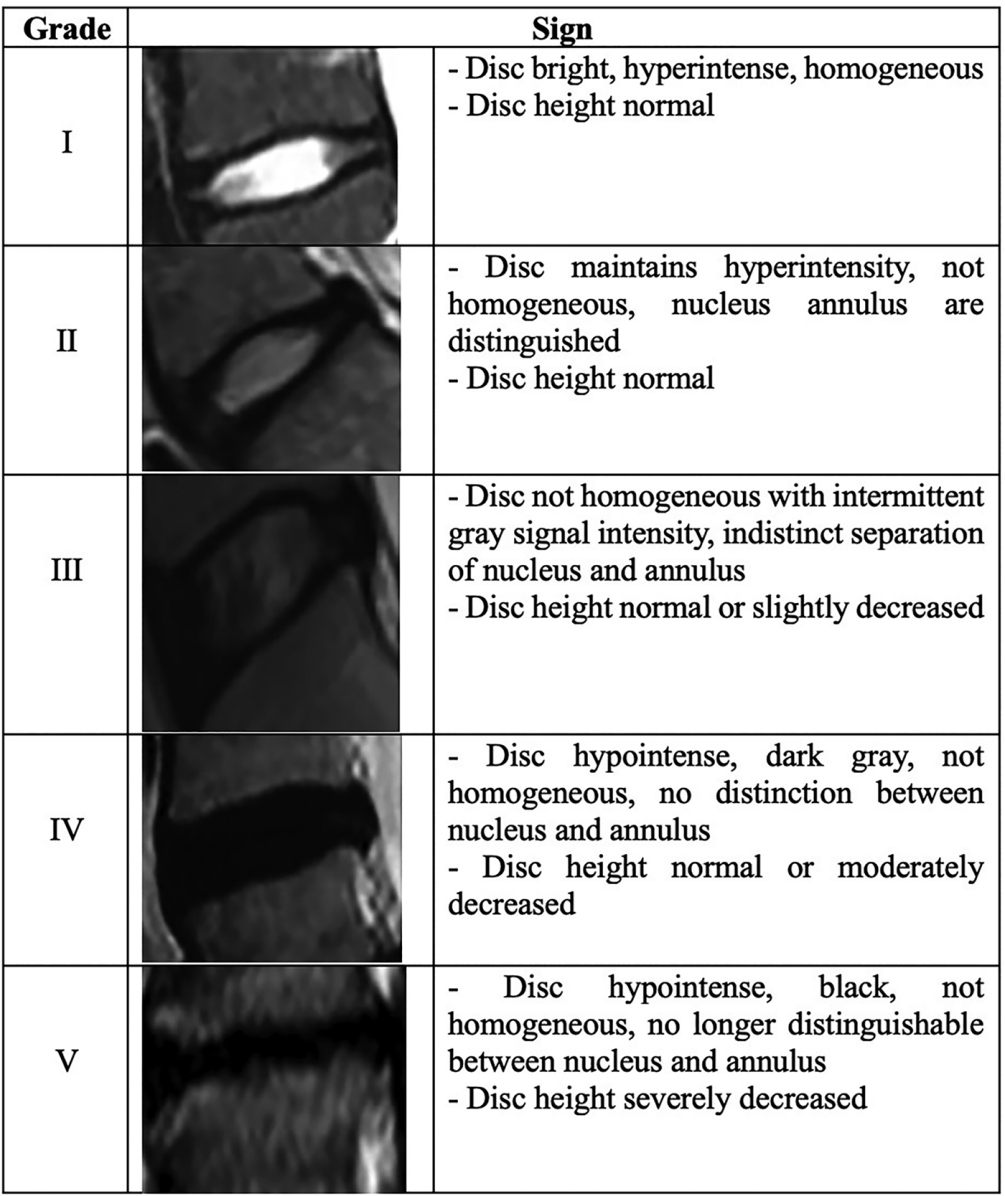
Pfirrmann grading system.

All patients underwent a 1.5-T MRI scan (MAGNETOM Avanto, Siemens Healthcare, Forchheim, Germany) for comprehensive evaluation. The imaging protocol included sagittal T1-weighted, sagittal T2-weighted, and axial T2-weighted sequences, each acquired with the following parameters: FOV, 350 mm; slice thickness, 3.5 mm; voxel size, 0.8 mm × 0.8 mm × 3.5 mm. Postoperative MRI examinations were conducted at an average interval of 42 months (range: 30–136), allowing for a thorough assessment of changes over time. Utilizing the Pfirrmann classification, degrees of adjacent and implanted segment disc degeneration and regeneration were determined (14) (refer to Figure 2). In addition, preoperative anteroposterior and lateral standing x-rays of the lumbar spine were obtained for all patients, with follow-up x-rays conducted at 4, 12, and 24 months post-surgery.

**Figure 3:**
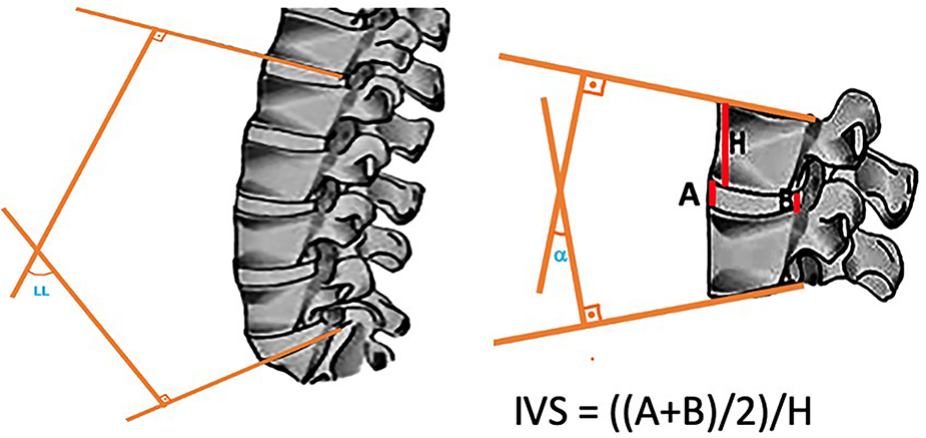
Two lines were drawn, one lateral to the lower end of the L1–L2 disc and the other lateral to the lower end of the L5–S1 disc, and the angle between them was determined as the lumbar lordosis angle. The segmental lordosis angle of the operative level was calculated as the angle of intersection between the line perpendicular to the line drawn on the upper end of the instrumented superior vertebra and the line perpendicular to the line drawn on the lower end of the inferior vertebral (α). The intervertebral disc height ratios determined by dividing the anterior disc height by the posterior disc height.

The lumbar lordosis angle was measured as the angle between the lines drawn lateral to the lower endplate of L1 and the upper endplate of S1. The segmental lordosis angle of the operative level was measured as the angle between the lines drawn on the superior endplate of the instrumented superior vertebra and the inferior endplate of the inferior vertebra. Intervertebral disc height ratios were calculated by dividing the anterior and posterior disc heights by the height of the rostral vertebra of the motion segment. All images were evaluated by a single experienced neuroradiologist (Figure 3).

### Clinical evaluation

The quality of life and pain levels of the patients were assessed by using the visual analog scale (VAS) and the Oswestry Disability Index (ODI) at multiple time points: preoperatively, postoperative fourth month, first year, and annually thereafter. In addition, body mass index (BMI) assessments were conducted at our clinic as part of the comprehensive evaluation process.

### Surgical method

Under general anesthesia, surgical procedures were performed with patients in the knee–chest position, strategically chosen to preserve lumbar lordosis. This positioning is particularly crucial for dynamic systems designed to address mobile deformities. In cases where waist movement correction could not be achieved with the table alone, Ponte or pedicle subtraction osteotomies were considered. The target operative segment was confirmed using fluoroscopic imaging.

A 3–4 cm midline skin incision was carefully made in accordance with the pathologic segment. Microsurgical decompression, involving laminectomy or laminotomy with microdiscectomy, was meticulously executed. The annular structure was then reconstructed using bipolar cauterization. Subsequently, employing the Wiltse approach, pedicle screws were precisely placed under fluoroscopic (C-arm) guidance (15). The lumbar lordosis angle was assessed before rod implantation, with efforts made to maintain its normal angle. The wound layers were meticulously closed with effective bleeding control. Patients were mobilized the day after surgery and typically discharged on the third or fourth postoperative day. A brief period of rest, approximately 1 month, was observed before patients resumed their normal daily activities.

### Statistical analysis

The normality of continuous variables was evaluated with the Shapiro–Wilk test. Descriptive statistics for continuous variables are expressed as mean ± standard deviation or median (interquartile range) depending on the normality of the distribution. According to variables, *t*-tests, Mann–Whitney *U*, one-way ANOVA, *χ*^2^, Fisher exact, and Friedman tests were applied. A *p*-value of <0.05 was considered statistically significant. Statistical analyzes were performed using jamovi software version 1.2.

## Results

The study cohort comprised 111 patients with a mean age of 50.2 ± 13.4 years and a median BMI of 28. The follow-up period ranged from 30 to 136 months, with a mean follow-up duration of 42 months. Significant differences were observed between preoperative and postoperative VAS and ODI scores (*p* < 0.001).

In terms of gender, operated segment-level distribution, and smoking rates, no significant differences were identified among the groups (*p* > 0.05). Moreover, no statistically significant variations were noted in terms of age, BMI, preoperative VAS, preoperative ODI, postoperative VAS, and postoperative ODI scores between the groups (*p* = 0.477, *p* = 0.477, *p* = 0.477, *p* = 0.693, *p* = 0.859, *p* = 0.742, *p* = 0.215, *p* = 0.067) (Table 1).

**Table 1:** Comparison of the ages, BMI, preoperative, and postoperative VAS and ODI of the three groups.

	Group	Age	BMI	Preop VAS	Preop ODI	Postop VAS	Postop ODI
*N*	Dynesys	38	38	38	38	38	38
	Saf + PEEK	37	37	37	37	37	37
	Saf + titan	36	36	36	36	36	36
Mean	Dynesys	48.2	27.3	7.53	69.1	1.03	8.05
	Saf + PEEK	50.7	27.8	7.43	70.2	1.08	8.22
	Saf + titan	51.9	28.1	7.42	68.8	1.25	9.94
Median	Dynesys	47.0	27.8	8.00	68.0	1.00	8.00
	Saf + PEEK	47	28.0	7	72	1	8
	Saf + titan	51.5	28.0	7.00	68.0	1.00	8.00
Standard deviation	Dynesys	11.5	4.10	0.922	9.58	0.592	3.56
	Saf + PEEK	15.1	3.97	0.987	6.23	0.595	4.92
	Saf + titan	13.3	3.95	0.874	8.19	0.500	2.55
IQR	Dynesys	13.3	5.60	1.00	6.00	0.00	4.00
	Saf + PEEK	18.0	6.00	1.00	10.0	0.00	6.00
	Saf + titan	20.8	6.00	1.00	11.0	1.00	4.00
Minimum	Dynesys	25	20.0	5	56	0	2
	Saf + PEEK	23	20.0	5	58	0	2
	Saf + titan	26	20.0	6	52	0	6
Maximum	Dynesys	77	35.0	9	92	2	18
	Saf + PEEK	86	35.0	9	82	2	26
	Saf + titan	77	36.0	9	92	2	16

No significant distribution differences were observed between the groups in terms of preoperative/postoperative Pfirrmann disc degeneration/regeneration score changes at the operated level and adjacent segments (*p* = 0.763, *p* = 0.518, *p* = 0.201). Disc regeneration percentages at the operated level were 31.6%, 35.1%, and 30.6%, while degeneration percentages were 15.8%, 10.8%, and 19.4% for the respective groups. Importantly, the disc distance remained consistent at the operated, upper adjacent, and lower adjacent levels (Tables 2–4).

**Table 2:** Statistical comparison of the disc regeneration, degeneration, and preserved disc ratios of each of the three groups in the operation segment.

	Groups			
O	Dynesys	Saf + PEEK	Saf + titan	Total
−2				
Observed	0	1	0	1
% within column	0.0%	2.7%	0.0%	0.9%
−1				
Observed	6	3	7	16
% within column	15.8%	8.1%	19.4%	14.4%
0				
Observed	20	20	18	58
% within column	52.6%	54.1%	50.0%	52.3%
1				
Observed	12	13	11	36
% within column	31.6%	35.1%	30.6%	32.4%
Total				
Observed	38	37	36	111
% within column	100.0%	100.0%	100.0%	100.0%

**Table 3:** Statistical comparison of the disc degeneration and preserved disc ratios of each of the three groups in the superior adjacent segment.

Superior adjacent segment	Dynesys	Saf + PEEK	Saf + titan	Total
−3				
Observed	1	0	2	3
% within column	2.6%	0.0%	5.6%	2.7%
−2				
Observed	1	1	0	2
% within column	2.6%	2.7%	0.0%	1.8%
−1				
Observed	1	4	3	8
% within column	2.6%	10.8%	8.3%	7.2%
0				
Observed	35	32	31	98
% within column	92.1%	86.5%	86.1%	88.3%
Total				
Observed	38	37	36	111
% within column	100.0%	100.0%	100.0%	100.0%

**Table 4:** Statistically comparison of the disc degeneration and preserved disc ratios of each of the three groups in the inferior adjacent segment.

Inferior adjacent segment	Dynesys	Saf + PEEK	Saf + titan	Total
−2				
Observed	1	0<	1	2
% within column	4.5%	0.0%	4.3%	2.7%
−1				
Observed	3	1	4	8
% within column	13.6%	3.3%	17.4%	10.7%
0				
Observed	18	29	18	65
% within column	81.8%	96.7%	78.3%	86.7%
Total				
Observed	22	30	23	75
% within column	100.0%	100.0%	100.0%	100.0%

No statistically significant differences were detected between the groups concerning changes in lumbar and segmental lordosis angles and intervertebral disc height ratios, as observed in anteroposterior and lateral radiographs of the standing lumbar spine conducted at preoperative, fourth, 12th, and 24th months (*p* > 0.05). Within each group, no significant differences were seen in the changes of these parameters over time (*p* > 0.05). Other spinopelvic parameters were normal or close to normal values in all cases.

A notable finding was the statistically significant difference in preoperative intervertebral disc height ratios, exclusively identified between the Dynesys and Safinaz–titanium groups (*p* = 0.029) (Table 5). Furthermore, no statistically significant differences were observed between the groups in terms of operation rates related to adjacent segment disease, screw breakage, and loosening during postoperative follow-up (*p* > 1.000, *p* = 0.655, *p* > 1.000) (Table 6).

**Table 5:** Statistically comparison of the lumbar and segmental lordosis angles and the changes in the intervertebral disc heights in each of the three groups.

	Dynesys		Safinaz–PEEK		Safinaz–titanium				
	Mean ± SD	Median (min–max)	Mean ± SD	Median (min–max)	Mean ± SD	Median (min–max)	Test value	*p*	*Post hoc*
Lumbar lordosis angle preop	42.31 ± 11.88	44 (19–67)	45.16 ± 11.87	44 (17–64)	43.02 ± 10.38	43.5 (25–64)	0.627	0.536	
Lumbar lordosis angle fourth month	42.39 ± 9.78	43.5 (22–63)	45.29 ± 10.66	45 (18–61)	42.61 ± 11.1	42.5 (18–64)	0.876	0.419	
Lumbar lordosis angle 12th month	42.73 ± 9.77	42.5 (22–61)	45.18 ± 11.14	45 (16–62)	42.5 ± 11.02	42 (17–61)	0.721	0.489	
Lumbar lordosis angle 24th month	42.71 ± 9.77	42.5 (23–60)	45.27 ± 11.17	45 (16–65)	43.08 ± 10.49	43 (23–63)	0.645	0.526	
Test value	0.123		0.018		0.374				
*p*	0.816		0.977		0.664				
Segmental lordosis angle preop	26.63 ± 10.08	26.5 (10–42)	24.02 ± 7.27	24 (6–38)	25.52 ± 7.31	25 (12–41)	0.916	0.403	
Segmental lordosis angle fourth month	26.55 ± 8.59	26.5 (9–40)	24.51 ± 7.84	25 (7–45)	25.08 ± 6.8	24.5 (12–38)	0.684	0.507	
Segmental lordosis angle 12th month	26.42 ± 8.7	27 (9–40)	24.7 ± 7.52	26 (7–39)	25.3 ± 7.35	24 (13–42)	0.458	0.634	
Segmental lordosis angle 24th month	26.97 ± 9.05	28 (10–40)	24.59 ± 7.37	26 (7–38)	25.36 ± 6.82	24.5 (13–41)	0.903	0.408	
Test value	0.180		0.464		0.266				
*p*	0.829		0.620		0.764				
Intervertebral disc rate preop	0.29 ± 0.06	0.29 (0.18–0.45)	0.27 ± 0.07	0.26 (0.09–0.39)	0.25 ± 0.06	0.24 (0.12–0.39)	3.658	**0.029** *	Dyn–Saf T
Intervertebral disc rate fourth month	0.3 ± 0.06	0.3 (0.18–0.4)	0.28 ± 0.07	0,27 (0.12–0.42)	0.27 ± 0.07	0.27 (0.11–0.42)	1.551	0.217	
Intervertebral disc rate 12th month	0.29 ± 0.06	0.29 (0.14–0.41)	0.27 ± 0.05	0,27 (0.1–0.39)	0.26 ± 0.06	0.25 (0.09–0.37)	1.875	0.158	
Intervertebral disc rate 24th month	0.29 ± 0.06	0.29 (0.15–0.41)	0.27 ± 0.05	0.27 (0.12–0.37)	0.26 ± 0.06	0.25 (0.14–0.38)	2.054	0.133	
Test value	0.433		0.913		1.232				
*p*	0.666		0.411		0.298				

* *p* < 0.05.

**Table 6:** Statistical comparison of the ratios of screw loosening, breakage, and adjacent segment disease development in the postoperative period and the ratios of surgeries performed due to these among the three groups.

Screw loosening					
	Dynesys	Safinaz	Safinaz + titanium	Test value	*p*
Present	1 (2.63%)	1 (2.7%)	1 (2.78%)	0.434	1.000
Absent	37 (97.37%)	36 (97.3%)	35 (97.22%)		
	38	37	36		
Screw breakage					
	Dynesys	Safinaz	Safinaz + titanium	Test value	*p*
Present	2 (5.26%)	0 (0%)	1 (2.78%)	1.820	0.655
Absent	36 (94.74%)	37 (100%)	35 (97.22%)		
	38	37	36		
Adjacent segment disease					
	Dynesys	Safinaz	Safinaz + titanium	Test value	*p*
Present	2 (5.26%)	2 (5.41%)	1 (2.78%)	0.551	1.000
Absent	36 (94.74%)	35 (94.59%)	35 (97.22%)		
	38	37	36		
Loosening + breakage + operations due to adjacent segment disease					
	Dynesys	Safinaz	Safinaz + titanium	Test value	*p*
Present	5 (13.16%)	3 (8.11%)	3 (8.33%)	0.700	0.786
Absent	33 (86.84%)	34 (91.89%)	33 (91.67%)		
	38	37	36		

**Figure 4:**
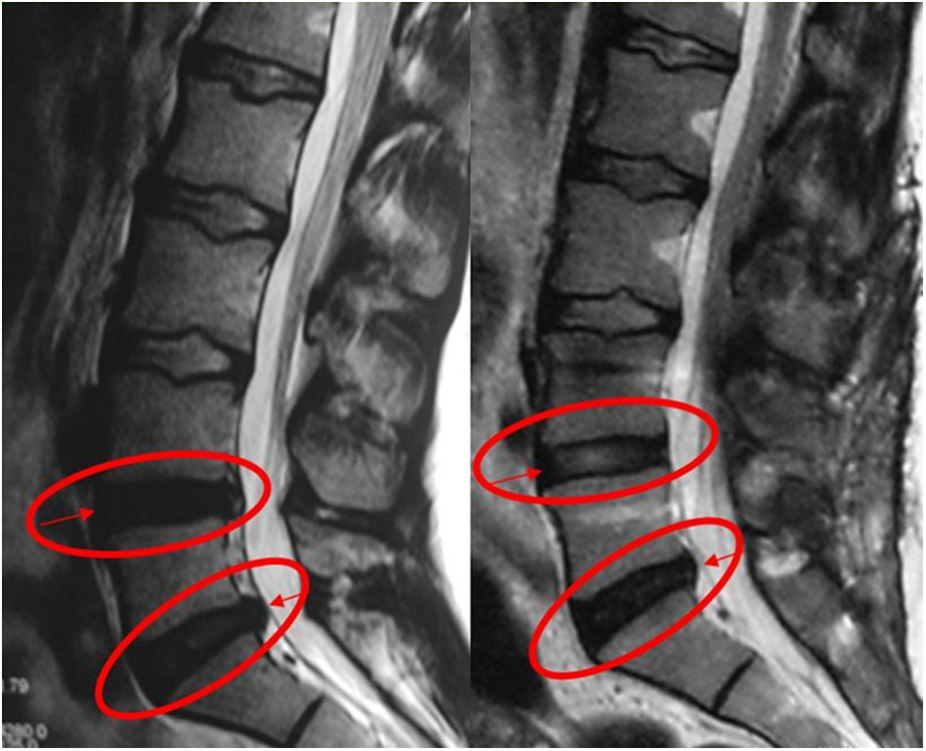
The patient with painful degenerative disc disease was grade 4 according to Pfirrmann classification due to decreased signal intensity in the preoperative T2-weighted MRI. After dynamic stabilization with Safinaz screw and PEEK rod, the patient was evaluated as grade 3 (L4–L5) due to the increase in intensity in the T2-weighted MRI taken at the 12th month. No change in signal intensity at the L5–S1 level after 12 months postoperatively.

**Figure 5:**
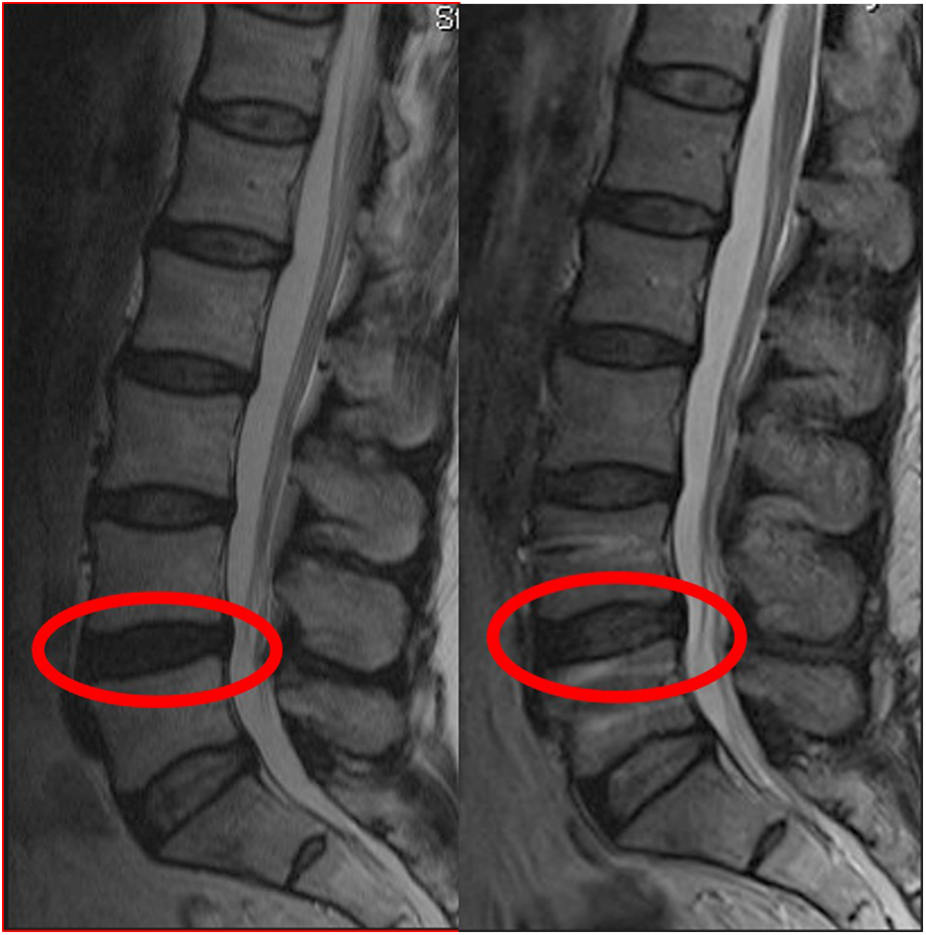
The patient with painful degenerative disc disease was grade 4 according to Pfirrmann classification due to decreased signal intensity in the preoperative T2-weighted MRI. After dynamic stabilization with Dynesys, the patient was evaluated as grade 3 due to the increase in intensity in the T2-weighted MRI taken at the 12th month.

**Figure 6:**
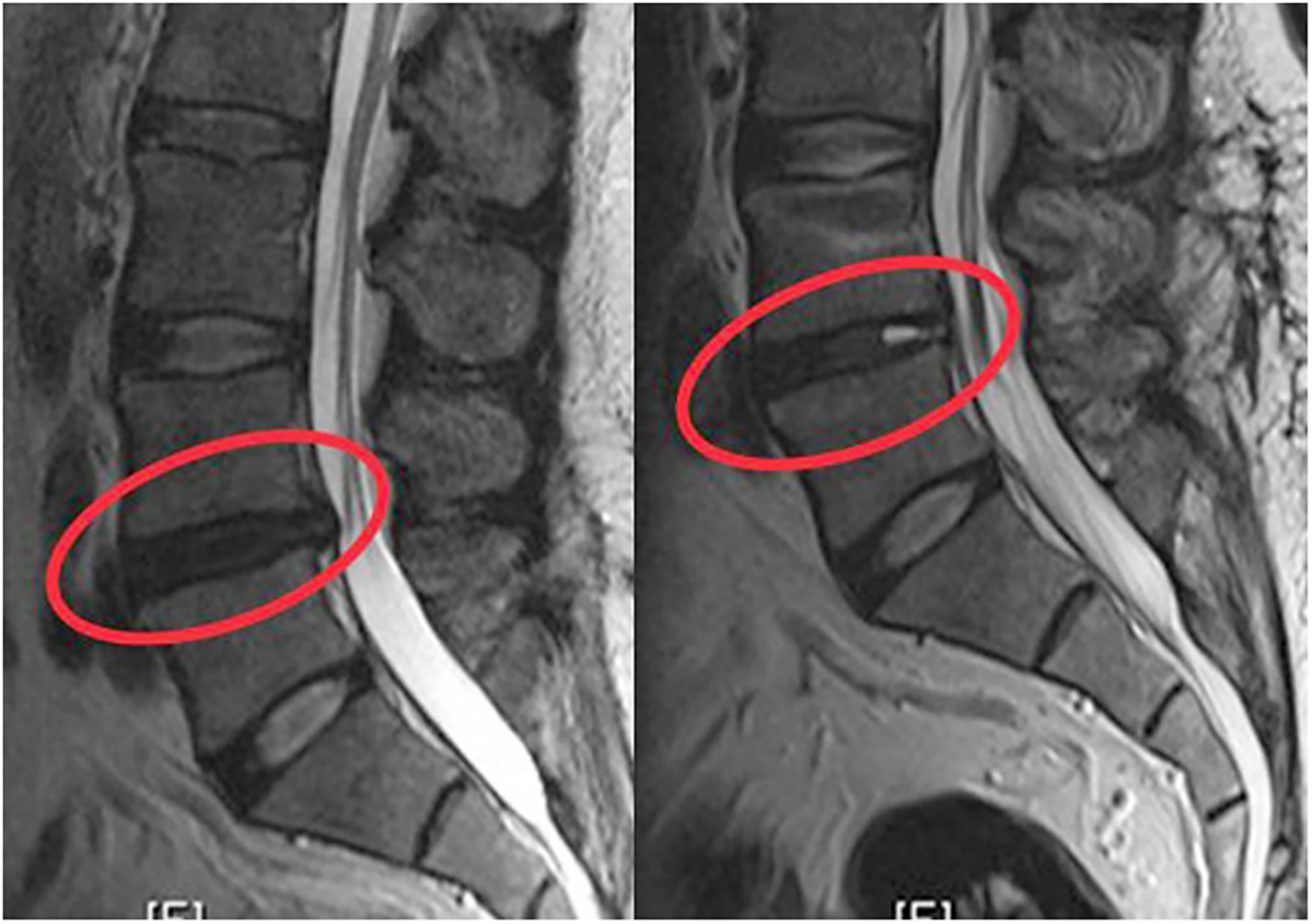
The patient with painful degenerative disc disease was grade 4 according to Pfirrmann classification due to decreased signal intensity in the preoperative T2-weighted MRI. After dynamic stabilization with Safinaz screw and titanium rod, the patient was evaluated as grade 3 due to the increase in intensity in the T2-weighted MRI taken at the 12th month.

No more than 300 ml of blood was lost during the operation. Operation times range from 60 to 100 min. No mortality or morbidity was observed in the peri- or postoperative period. Illustrative cases from this series are provided in Figures 4–6.

## Discussion

In this study, clinical and radiological results of three different transpedicular dynamic systems, which have been preferred as an alternative to fusion surgery in the treatment of degenerative disc diseases, are presented. PEEK rod (CD–Horizon Legacy; Medtronic Sofamor Danek) provides less rigidity in the spine compared with metal systems and more rigidity than the Dynesys system; therefore, it reduces the rate of screw loosening and breakage and increases fusion rates (16, 17). Biomechanical studies have shown that a new concept, dynamic rod and dynamic (hinged) screw, restores the unstable segment (18, 19).

Fusion surgery with decompression has been preferred for more than 30 years in the treatment of degenerative disc diseases. It is believed that abnormal movements of the degenerated segment cause pain and the fusion of this segment will relieve the pain. In the follow-ups after fusion surgery, degeneration in the adjacent segment has been reported (12, 20). In a recent study, the incidence of radiological adjacent segment degeneration was found to be 32.8% after lumbar fusion surgery. Moreover, approximately one-fourth to one-third of this radiological degeneration progressed to adjacent segment disease (13). According to another study, the rate of degeneration development in the adjacent segment was 16.5% in the first 5 years; the result was 36.1% in the first 10 years (21). In our study, radiological disc degeneration in the superior adjacent segment was observed in 13 of 111 (11.7%) patients, and radiological disc degeneration in the inferior adjacent segment was observed in 10 of 75 (13.33%) patients. Five (4.5%) of our patients were operated for adjacent segment disease.

Among the dynamic systems, Dynesys is the most widely used one in the world. This system was developed against the disadvantages of fusion surgery by Dubois in 1994. In this system, an inelastic artificial tension band is placed between the pedicle screws, and a polyurethane tube is placed around the band. Stabilizing the posterior elements in this way reduces the load on the facet joints and discs and partially preserves the motion of the relevant segment (22). Thus, studies have shown that Dynesys system prevents the degeneration of adjacent segments (10). In addition, it has been shown in the literature that satisfactory results can be obtained when compared with fusion systems (23–25). However, the fact that the Dynesys system is not recommended for use in trauma, total facetectomy patients, isthmus fractures, and high-grade spondylolisthesis is among its disadvantages (26). In addition, long preparation of the spacers during surgery causes kyphosis, which also causes an increase in pressure in the anterior compartment. Compressive loads can cause pedicular rotation, resulting in screw breakage and loosening (27).

In the study by Stoll et al., 83 patients were treated with Dynesys, two patients were reoperated due to screw loosening, one patient was reoperated due to root compression of the screw, and seven patients were reoperated due to development of adjacent segment disease (27). The Oswestry score decreased from 55.4% preoperatively to 22.9% postoperatively. The authors suggested that Dynesys is less invasive and has a low rate of adjacent segment degeneration.

Schaeren et al. (28) reported a study of 26 patients treated with Dynesys. Screw loosening was observed in three patients, 2 years after the operation, but no surgical intervention was performed. Four years later, instability due to screw breakage was detected in one patient, and adjacent segment degeneration was detected in nine patients (47%).

Cakir et al. (29) investigated the adjacent segment mobility of the lumbar spine after rigid and semi-rigid instrumentation by dividing 26 patients into two groups. Decompression and posterior stabilization with Dynesys were performed in one group, and decompression and fusion surgery were performed in the other group. It was concluded that neither group had a beneficial effect on adjacent segment mobility.

In our study, adjacent segment degeneration developed in three (7.9%) of 38 patients who underwent Dynesys, and two were operated. In addition, screw loosening was observed in one patient, and screw breakage was observed in two patients. When the preoperative and postoperative VAS and ODI values were compared, a significant improvement was observed (Table 1).

Strempel (21, 30) was the first to articulate the screw in dynamic systems. The stability of the system is provided by a titanium rod, and its flexibility is provided by a pedicular screw system with an articulated neck. While allowing minimal movement in the sagittal plane, it does not allow rotation and translation. It distributes the load on the vertebra evenly between the anterior and posterior parts. Compared with the Dynesys system, the Cosmic system can be used in discogenic low back pain as well as in patients where laminectomy and total facetectomy have been performed. Moreover, it provides the restoration of lumbar lordosis (19, 21, 29).

The Safinaz screw system (Tıpsan, İzmir, Türkiye) we used in our study was developed based on the Cosmic system. Unlike the Cosmic screw, this screw is an articulated transpedicular system that allows flexion–extension of up to 20° and rotational motion of 2° (32). It has been shown to be effective when applied after discectomy in patients with low back pain due to Modic degeneration (31). In a cadaver study, it was revealed that stabilization was achieved close to the rigid system (18). However, due to the rigidity of the rod used in the system, it was reported that the system turned into a rigid structure in long segment stabilization cases. In the studies by Ozer et al., in which they compared the Safinaz system and the rigid system (fusion), it was observed that the sagittal balance was preserved with equivalent relief in both groups (16, 19, 31).

The problem is that you can make the system somewhat flexible with a Ti rod by putting a dynamic screw. The flexibility can be increased a little more using the PEEK rod. In order to make the system even more flexible, you need to stretch the rod more. In our cadaveric studies, when we stretched the rod further, we saw that the movements of the impaired motion segment reached physiological limits. In fact, we used all kinds of dynamic rods in the market (Agile Rod, Balance C Rod Medtronic, and Dream Rod CE–certified Korean Rod) with dynamic screws, but all of them broke in an average of 4 years (not in a year or two as in the rigid systems), and they turned out to be insufficient. After all, no flexible rod was available in the market other than Dynesys and maybe a PEEK rod that we can call a little flexible.

Rod flexibility is the most open-to-criticism aspect of dynamic systems. No standardization is noted. In fact, no universally accepted dynamic rod is currently available in the market. PEEK rod features are standard but not ideal. The complications in our case arise from the dynamic system not being ideal, that is, the movement segment remaining more rigid than its ideal movements or the insufficiency of bone stock in the patient.

Kaner et al. (32) reported the results of their study in which they applied Cosmic and rigid transpedicular stabilization systems. In both groups, VAS and ODI were scanned; radiological disc height, lumbar lordosis, and segmental lordosis angle measurements were made; and similar results were obtained.

Ozer et al. (16) reported the results of a study in which they applied dynamic stabilization and rigid fixation system in 41 patients. During the follow-up period, the disc height ratios and the lumbar and segmental lordosis angles remained unchanged in both groups.

In our study, no statistically significant change was observed in the lumbar lordosis angle, segmental lordosis angle, and intervertebral disc height ratios in all three groups. In addition, in our study, rates of adjacent segment degeneration were found to be quite low in the Safinaz and PEEK rod groups compared with the other groups (Tables 3, 4).

It has been suggested that dynamic stabilization systems protect the physiological movement and intradisc pressure in the operated segment with axial distraction and thus prevent degeneration, as well as provide regeneration of the disc tissue when suitable conditions are provided (5, 33). It has been reported that dynamic systems prevent the progression of degeneration in the operated segment (34). Cho et al. published a case report showing obvious disc regeneration in which they applied dynamic stabilization (35). In their study, Zhang et al. reported that no significant clinical improvement was reported in terms of disc rehydration and disc degeneration in patients who underwent dynamic stabilization (36). This article offers a clear perspective on the conservation of the movement. According to Pfirrmann criteria, no preservation of motion by using dynamic systems in advanced degeneration and fusion should be made. However, we can say that disc motion can be preserved with dynamic systems in the early stage of disc degeneration.

In our study, significant clinical improvement was observed in VAS and ODI scores in all groups which were followed up for 42 months. In the postoperative MRI, when the operated segment was evaluated according to Pfirrmann, 58 (52.3%) patients showed no change, 36 (32.4%) showed regeneration, and 17 (15.3%) showed degeneration. From this, it has been seen that all three dynamic systems maintain or improve the disc distance in their current form at close ratios to each other (Table 2). However, Pfirrmann classification is just a morphological/radiological imaging classification without any clinical correlation, and the observation of a postop disc rehydration does not mean regeneration. In addition to the improvement in the Pfirrmann grade, the clinical recovery of the patients is one of the most important data. In the literature, the relationship between disc degeneration and the rate of gadolinium influx measured through MRI has been investigated. The authors developed a total endplate score (TEPS) and suggested that biologic therapies will only succeed in discs with a TEPS of 6 (on a scale of 1–12). However, this TEPS approach has not yet been independently validated.

It has been reported that the incidence of implant failure, such as screw loosening or breakage, varies between 2.6% and 36% after fusion surgery (37). Screw loosening is one of the most frequently reported complications following Dynesys stabilization (38). In a previous long-term follow-up study, it was reported that screw loosening was 20.5% (22 of 107 patients) and three patients underwent reoperation (39). In our study, in the Dynesys group, screw loosening was found in one patient (2.63%), and screw breakage was found in two patients (5.26%), while two patients (5.26%) were reoperated due to the development of adjacent segment disease. In the Safinaz and PEEK rod groups, screw loosening was detected in one patient (2.7%), and two patients (5.41%) were reoperated due to the development of adjacent segment disease. In the Safinaz and titanium rod groups, screw breakage was found in one patient (2.7%), screw loosening was found in one patient (2.78%), and one patient (2.78%) was reoperated for adjacent segment disease. Postoperative infection was observed in five patients (4.5%), and the patients were treated with antibiotic administration. Discitis was detected in one patient.

We developed an algorithm to improve our surgical results in patients to whom we applied a dynamic system. We evaluate T scores of the patients. We perform the surgery in two stages in patients with a T score of above −1.5. In the first stage, we put the screws and wait for osteointegration to solidify, and we do the second stage after 4 months, perform decompression, and place the rods. In this way, in elderly patients with poor bone quality, we prevent screw from loosening (40).

## Conclusion

It has been observed that the degeneration rates of adjacent segments and the implanted segment may decrease, and the operated segment may be regenerated in patients who are not severely degenerative. In addition, it was observed that the lumbar lordosis angle, segmental lordosis angle, and disc height were preserved. Even in severe degenerative disc diseases, it has been shown that the dynamic system can offer a better quality of life by limiting micro-instability. Dynamic systems should be a preferred surgical technique because of the short duration of hospitalization, ease of application, and low complication rates.

## Data Availability

The original contributions presented in the study are included in the article/Supplementary Material, further inquiries can be directed to the corresponding author.

## References

[1] Davis JR. Dynesys dynamic stabilisation system. In: Yue JJ Bertagnoli R McAfee PC An HS, editors. Motion preservation surgery of the spine. Philadelphia: Elsevier (2008). p. 465–71.

[2] Gilgil E Kacar C Bütün B Tuncer T Urhan S Yildirim C Prevalence of low back pain in a developing urban setting. Spine. (2005) 30(9):1093–8. 10.1097/01.brs.0000161007.46849.4c 15864165

[3] Hickey DS Hukins DW. X-ray diffraction studies of the arrangement of collagenous fibres in human fetal intervertebral disc. J Anat. (1980) 131(Pt 1):81–90. 7440405 PMC1233288

[4] Zhao CQ Wang LM Jiang LS Dai LY. The cell biology of intervertebral disc aging and degeneration. Ageing Res Rev. (2007) 6:247–61. 10.1016/j.arr.2007.08.001 17870673

[5] Yilmaz A Senturk S Sasani M Oktenoglu T Yaman O Yildirim O Disc rehydration after dynamic stabilization: a report of 59 cases. Asian Spine J. (2017) 11(3):348–55. 10.4184/asj.2017.11.3.348 28670402 PMC5481589

[6] Roberts S Evans H Trivedi J Menage J. Histology and pathology of the human intervertebral disc. J Bone Joint Surg Am. (2006) 88(Annex 2):10–4. 10.2106/JBJS.F.00019 16595436

[7] Fritzell P Hägg O Wessberg P Nordwall A; Swedish Lumbar Spine Study Group. Chronic low back pain and fusion: a comparison of three surgical techniques: a prospective multicenter randomized study from the Swedish lumbar spine study group. Spine. (2002) 27:1131–42. 10.1097/00007632-200206010-00002 12045508

[8] Oktenoglu T Ozer AF Sasani M Kaner T Canbulat N Ercelen O Posterior dynamic stabilization in the treatment of lumbar degenerative disc disease: 2-year follow-up. Minim Invasive Neurosurg. (2010) 53(3):112–6. 10.1055/s-0030-1262810 20809451

[9] Ozer AF Oktenoglu T Egemen E Sasani M Yilmaz A Erbulut DU Lumbar single-level dynamic stabilization with semi-rigid and full dynamic systems: a retrospective clinical and radiological analysis of 71 patients. Clin Orthop Surg. (2017) 9(3):310–6. 10.4055/cios.2017.9.3.310 28861198 PMC5567026

[10] Würgler-Hauri CC Kalbarczyk A Wiesli M Landolt H Fandino J. Dynamic neutralization of the lumbar spine after microsurgical decompression in acquired lumbar spinal stenosis and segmental instability. Spine. (2008) 33(3):E66–72. 10.1097/BRS.0b013e31816245c0 18303447

[11] Boos N Webb JK. Pedicle screw fixation in spinal disorders: a European view. Eur Spine J. (1997) 6:2–18. 10.1007/BF01676569 9093822 PMC3454634

[12] Harrop JS Youssef JA Maltenfort M Vorwald P Jabbour P Bono CM Lumbar adjacent segment degeneration and disease after arthrodesis and total disc arthroplasty. Spine. (2008) 33(15):1701–7. 10.1097/BRS.0b013e31817bb956 18594464

[13] Hashimoto K Aizawa T Kanno H Itoi E. Adjacent segment degeneration after fusion spinal surgery: a systematic review. Int Orthop. (2019) 43:987–93. 10.1007/s00264-018-4241-z 30470865

[14] Pfirrmann CW Metzdorf A Zanetti M Hodler J Boos N. Magnetic resonance classification of lumbar intervertebral disc degeneration. Spine. (2001) 26(17):1873–8. 10.1097/00007632-200109010-00011 11568697

[15] Wiltse LL Spencer CW. New uses and refinements of the paraspinal approach to the lumbar spine. Spine. (1988) 13(6):696–706. 10.1097/00007632-198813060-00019 3175760

[16] Ozer AF Crawford NR Sasani M Oktenoglu T Bozkus H Kaner T Dynamic lumbar pedicle screw-rod stabilization: 2-year follow-up and comparison with fusion. Open Orthop J. (2010) 4:137–41. 10.2174/1874325001004010137 20448815 PMC2864427

[17] Ormond DR Albert L Jr Das K. Polyetheretherketone (PEEK) rods in lumbar spine degenerative disease: a case series. Clin Spine Surg. (2016) 29(7):E371–5. 10.1097/BSD.0b013e318277cb9b 23075859

[18] Oktenoglu T Erbulut DU Kiapour A Ozer AF Lazoglu I Kaner T Pedicle screw-based posterior dynamic stabilisation of the lumbar spine: in vitro cadaver investigation and a finite element study. Comput Methods Biomech Biomed Eng. (2015) 18(11):1252–61. 10.1080/10255842.2014.890187 24708377

[19] Bozkus H Senoglu M Baek S. Dynamic lumbar pedicle screw-rod stabilization: in vitro biomechanical comparison with standard rigid pedicle screw-rod stabilization-laboratory investigation. J Neurosurg Spine. (2010) 12(2):183–9. 10.3171/2009.9.SPINE0951 20121354

[20] Akyüz ME Firidin MN. Adjacent segment degeneration following spinal fusion for degenerative lumbar disease: incidence and risk factors. Uludağ Üniversitesi Tıp Fakültesi Dergisi. (2022) 48(2):225–9. 10.32708/uutfd.1130154

[21] Strempel A. Cosmic: dynamic stabilization of the degenerated lumbar spine. In: Yue JJ Bertagnoli R McAfee PC An HS, editors. Motion preservation surgery of the spine. Philadelphia: Elsevier (2008). p. 490–9.

[22] Dubois G de Germay B Schaerer NS Fennema P. Dynamic neutralization: a new concept for restabilization of the spine. In: Szpalski M Gunzburg R Pope MH, editors. Lumbar segmental instability. Philadelphia, PA: Lippincott Williams and Wilkins (1999). p. 233–40.

[23] Wu H Pang Q Jiang G. Medium-term effects of Dynesys dynamic stabilization vs. posterior lumbar interbody fusion for treatment of multisegmental lumbar degenerative disease. J Int Med Res. (2017) 45:1562–73. 10.1177/0300060517708104 28661265 PMC5718723

[24] Bredin S Demay O Mensa C Madi K Ohl X. Posterolateral fusion vs. Dynesys dynamic stabilization: retrospective study at a minimum 5.5 years’ follow-up. Orthop Traumatol Surg Res. (2017) 103:1241–4. 10.1016/j.otsr.2017.07.020 28942026

[25] Schmoelz W Onder U Martin A von Strempel A. Non-fusion instrumentation of the lumbar spine with a hinged pedicle screw rod system: an in vitro experiment. Eur Spine J. (2009) 18(10):1478–85. 10.1007/s00586-009-1052-3 19504129 PMC2899377

[26] Bono CM Kadaba M Vaccaro AR. Posterior pedicle fixation based dynamic stabilization devices for the treatment of degenerative diseases of the lumbar spine. J Spinal Disord Tech. (2009) 22(5):376–83. 10.1097/BSD.0b013e31817c6489 19525796

[27] Stoll TM Dubois G Schwarzenbach O. The dynamic neutralization system for the spine: a multi-center study of a novel non-fusion system. Eur Spine J. (2002) 11(Suppl 2):S170–8. 10.1007/s00586-002-0438-2 12384741 PMC3611570

[28] Schaeren S Broger I Jeanneret B. Minimum four-year follow-up of spinal stenosis with degenerative spondylolisthesis treated with decompression and dynamic stabilization. Spine. (2008) 33(18):E636–42. 10.1097/BRS.0b013e31817d2435 18708915

[29] Cakir B Carazzo C Schmidt R Mattes T Reichel H Käfer W. Adjacent segment mobility after rigid and semirigid instrumentation of the lumbar spine. Spine. (2009) 34(12):1287–91. 10.1097/BRS.0b013e3181a136ab 19455004

[30] Strempel A. Nonfusion stabilization of the degenerated lumbar spine with cosmic. In: Kim DH Cammisa FP Jr Fessler RG, editors. Dynamic reconstruction of the spine. New York, NY: Thieme Medical Publishers. Inc. (2006). p. 330–9.

[31] Eser O Gomleksiz C Sasani M Oktenoglu T Aydin AL Ataker Y Dynamic stabilisation in the treatment of degenerative disc disease with Modic changes. Adv Orthop. (2013) 2013:806267. 10.1155/2013/806267 23781343 PMC3671504

[32] Kaner T Dalbayrak S Oktenoglu T Sasani M Aydin AL Ozer FO. Comparison of posterior dynamic and posterior rigid transpedicular stabilization with fusion to treat degenerative spondylolisthesis. Orthopedics. (2010) 33(5):33–8. 10.3928/01477447-20100329-09 20506953

[33] Schnake KJ Putzier M Haas NP Kandziora F. Mechanical concepts for disc regeneration. Eur Spine J. (2006) 15(Suppl 3):S354–60. 10.1007/s00586-006-0176-y 16835733 PMC2335380

[34] Schnake KJ Schaeren S Jeanneret B. Dynamic stabilization in addition to decompression for lumbar spinal stenosis with degenerative spondylolisthesis. Spine. (2006) 31:442–9. 10.1097/01.brs.0000200092.49001.6e 16481955

[35] Cho BY Murovic J Park KW Park J. Lumbar disc rehydration postimplantation of a posterior dynamic stabilization system. J Neurosurg Spine. (2010) 13:576–80. 10.3171/2010.5.SPINE08418 21039146

[36] Zhang Y Zhang ZC Li F Sun TS Shan JL Guan K Long-term outcome of Dynesys dynamic stabilization for lumbar spinal stenosis. Chin Med J. (2018) 131(21):2537–43. 10.4103/0366-6999.244107 30381586 PMC6213831

[37] Mohi Eldin MM Ali AM. Lumbar transpedicular implant failure: a clinical and surgical challenge and its radiological assessment. Asian Spine J. (2014) 8(3):281–97. 10.4184/asj.2014.8.3.281 24967042 PMC4068848

[38] Pham MH Mehta VA Patel NN Jakoi AM Hsieh PC Liu JC Complications associated with the Dynesys dynamic stabilization system: a comprehensive review of the literature. Neurosurg Focus. (2016) 40:E2. 10.3171/2015.10.FOCUS15432 26721576

[39] Sapkas G Mavrogenis AF Starantzis KA Soultanis K Kokkalis ZT Papagelopoulos PJ Outcome of a dynamic neutralization system for the spine. Orthopedics. (2012) 35:e1497–502. 10.3928/01477447-20120919-19 23027487

[40] Ozer AF Basak AT Ozbek MA Hekimoglu M Aydin AL Ates O Lumbar dynamic stabilization with 2-stage surgery: early results. Int J Spine Surg. (2022) 16(4):638–45. 10.14444/8306 35728831 PMC9421274

